# Phylogenetic and Geospatial Evidence of Canine Parvovirus Transmission between Wild Dogs and Domestic Dogs at the Urban Fringe in Australia

**DOI:** 10.3390/v12060663

**Published:** 2020-06-19

**Authors:** Mark Kelman, Lana Harriott, Maura Carrai, Emily Kwan, Michael P. Ward, Vanessa R. Barrs

**Affiliations:** 1 Sydney School of Veterinary Science, The University of Sydney, Sydney, NSW 2006, Australia; maura.carrai@sydney.edu.au (M.C.); emilypkwan2.7@gmail.com (E.K.); michael.ward@sydney.edu.au (M.P.W.); vanessa.barrs@cityu.edu.hk (V.R.B.); 2 Pest Animal Research Centre, Biosecurity Queensland, Department of Agriculture and Fisheries, Toowoomba, QLD 4350, Australia; Lana.Harriott@daf.qld.gov.au; 3 Jockey Club College of Veterinary Medicine, City University of Hong Kong, Kowloon Tong, Hong Kong, China

**Keywords:** canine parvovirus, peri-urban, wild dogs, disease transmission, Australia

## Abstract

Canine parvovirus (CPV) is an important cause of disease in domestic dogs. Sporadic cases and outbreaks occur across Australia and worldwide and are associated with high morbidity and mortality. Whether transmission of CPV occurs between owned dogs and populations of wild dogs, including *Canis familiaris*, *Canis lupus dingo* and hybrids, is not known. To investigate the role of wild dogs in CPV epidemiology in Australia, PCR was used to detect CPV DNA in tissue from wild dogs culled in the peri-urban regions of two Australian states, between August 2012 and May 2015. CPV DNA was detected in 4.7% (8/170). There was a strong geospatial association between wild-dog CPV infections and domestic-dog CPV cases reported to a national disease surveillance system between 2009 and 2015. Postcodes in which wild dogs tested positive for CPV were 8.63 times more likely to also have domestic-dog cases reported than postcodes in which wild dogs tested negative (*p* = 0.0332). Phylogenetic analysis of CPV VP2 sequences from wild dogs showed they were all CPV-2a variants characterized by a novel amino acid mutation (21-Ala) recently identified in CPV isolates from owned dogs in Australia with parvoviral enteritis. Wild-dog CPV VP2 sequences were compared to those from owned domestic dogs in Australia. For one domestic-dog case located approximately 10 km from a wild-dog capture location, and reported 3.5 years after the nearest wild dog was sampled, the virus was demonstrated to have a closely related common ancestor. This study provides phylogenetic and geospatial evidence of CPV transmission between wild and domestic dogs in Australia.

## 1. Introduction

Canine parvovirus (CPV) is a single-stranded DNA virus belonging to the family *Parvoviridae*, subfamily *Parvovirinae*, genus *Protoparvovirus* and species *Carnivore Protoparvovirus 1* (CPPV-1) [1]. CPV infection occurs worldwide and has been reported in a range of carnivores, including domestic dogs and various wildlife species [2,3,4,5,6,7,8,9,10,11]. Viruses closely related to CPV include Feline parvovirus (FPV), Mink enteritis virus (MEV), Blue fox parvovirus (BFPV) and Racoon parvovirus (RPV) [12,13], so named due to their initial detection in these species. Clinical disease caused by *Carnivore protoparvovirus 1* in different host species ranges from asymptomatic to severe gastroenteritis, leukopenia, dehydration and death [14].

Although the exact origin of CPV remains unknown, CPV is thought to have evolved from FPV, or, more likely, that both viruses share a common ancestor that is likely associated with a wild carnivore host [12,13,15,16,17,18,19]. CPV poses a continuing threat for the emergence of new variants with pathogenic potential for multiple carnivore species in the order *Carnivora*.

Across Australia, CPV disease is relatively common in owned domestic dogs, particularly in rural and remote regions. An estimated 20,000 cases occur annually in Australia, with a reported incidence of 4.12 cases per 1000 dogs [20,21]. CPV infection has also been reported in wild canids, including four dingoes (*Canis lupus dingo*) (including a litter of three dingo puppies) and a dingo–dog hybrid, based on fecal antigen testing [10], as well as a red fox (*Vulpes vulpes*), based on serological testing [22]. However, the presence of CPV in Australian wild dogs has not been investigated. Wild and feral dogs are distributed throughout Australia, ranging from remote, isolated regions to peri-urban areas [23], though wild-dog numbers are not well documented [24].

In Australia, the term “wild dog” includes dingoes, feral domestic dogs (*Canis familiaris)* and hybrids [25]. The only other wild canid in Australia is the red fox, *Vulpes vulpes*, which was introduced as a result of European settlement [26]. The other feral carnivore in Australia is the feral cat, *Felis catus* [27], which is a likely parvovirus reservoir, given that FPV and CPV have been demonstrated in the Australian feral cat population [28,29,30]. Domestic dogs are also suggested as likely reservoir hosts for CPV in Africa, China and Mexico [31,32,33,34]. Furthermore, molecular investigations of CPV have provided evidence of cross-species bidirectional transmission between domestic dogs and wild carnivores [15,32]. While an epidemiological association between CPV case-occurrence in domestic dogs and geographical proximity to wild dogs and foxes in Australia has been reported [10], evidence of infection with the same or related strains of CPV is lacking. Peri-urban regions in Australia are a likely location to detect the presence of CPV in wild dogs, as there is close contact between human settlements and wild carnivore populations, which has been associated with exposure to CPV [32,35,36].

As well as being present in blood and feces during active infection, parvoviruses persist in mononuclear cells in peripheral blood or tissue [29,37] after fecal shedding of virus has ceased. This allows tissue samples to be used to detect both active and resolved infections in asymptomatic hosts that are no longer shedding virus in feces, and which likely represent latent infection, as reported for human parvoviruses [12,38]. CPV has been detected in wild carnivores and domestic cats, in a range of tissue samples after viral shedding has ceased, including blood, bone marrow, mesenteric lymph nodes, tongue, spleen and myocardium [12,29,37].

Our goal was to investigate the epidemiology of parvoviruses at the wild–domestic dog interface. The specific objectives of this study were to determine whether wild-dog populations in Australia are exposed to CPV, and if so, to estimate its prevalence and identify circulating strains; and to assess the likelihood of transmission of CPV between wild dogs and domestic dogs in peri-urban regions.

## 2. Materials and Methods

### 2.1. Wild-Dog Sample Collection

The carcasses of 201 wild dogs captured and culled between August 2012 and May 2015 from peri-urban regions of South East Queensland (SEQ) and Northern New South Wales (NSW) were supplied by local councils, for a study investigating pathogens of public health or economic significance [24]. Ethics approval was granted by the University of Queensland AEC (Animal Ethics Committee) (SVS/145/13). Residual tissue samples (tongue), which had been stored frozen at −18 °C, were made available for CPV testing.

### 2.2. DNA Extraction and Conventional PCR

DNA was extracted from tongue tissue for PCR, using the Macherey-Nagel mini kit (Macherey-Nagel, Düren, Germany). To evaluate the presence and quality of canine DNA, a conventional PCR was performed, targeting a canine housekeeping gene, the ribosomal protein L32 (RPL32) gene, using the following primers: RPL32-F (5′- ACCTCTGGTGAAGCCCAAG-3′) and RPL32-R (5′- GGGATTGGTGACTCTGATGG-3′) [39]. The total reaction volume was 25 µL and contained 2.5 µL of template DNA, 0.5 µL each of forward- and reverse-primer, 0.5 µL of MyTaq^TM^HS Red DNA Polymerase, 5 µL of MyTaq ^TM^ Red Reaction Buffer and 16 µL of water. DNA amplification was performed by using an initial denaturation step at 95 °C for 1 min, followed by 35 cycles of 95 °C for 15 s, 60 °C for 15 s and 72 °C for 10 s, with a final extension step at 72 °C for 5 min. Following PCR, the samples were electrophoresed on a 1% agarose gel (Bio-Rad Laboratories, Hercules, CA, USA), using 1 × tris-borate EDTA running buffer, and visualized with SYBR safe DNA (Thermo Fisher Scientific, Waltham, MA, USA).

### 2.3. Real-Time PCR

Real-time PCR/quantitative PCR assay (qPCR) was performed to determine the CPV viral load in DNA extracts from tongue tissue of the wild-dog cadavers, as previously described, with minor modification [40]. In brief, real-time PCR was carried out in a 35 µL reaction containing 17.5 mL of IQ Supermix (Bio-Rad Laboratories Srl), 600 nM of primers CPV-For and CPV-Rev, 200 nM of probe CPV-Pb (Table 1), and 10 µL of template (diluted 1:10 in Tris EDTA buffer). Serial 8-fold dilutions (representing from 10^9^ to 10^2^ DNA copies/10 µL) of a plasmid, pFastBac^TM^HTA, containing VP2 gene sequence were used to generate a standard curve. Each test sample and each dilution of standard DNA was tested in duplicate. An exogenous DNA internal control, Cal Orange 560 (Bioline, Meridian Bioscience, Cincinnati, OH, USA), was added to each sample, in order to control for PCR inhibition, according to the manufacturer’s instructions. The thermal-cycle protocol used was the activation of Taq DNA polymerase at 95 °C for 10 min and 40 cycles consisting of denaturation at 95 °C for 15 s, primer annealing at 52 °C for 30 s and extension at 60 °C for 1 min. All reactions were conducted in an a CFX connect^TM^Real Time PCR Detection System (Bio-Rad Laboratories Pty., Ltd. Gladesville, Australia), and the data were analyzed with the software CFX Maestro. Samples were considered positive only when results could be confirmed from paired testing in a single assay.

### 2.4. Conventional PCR and Sequence Analysis

Samples testing positive for CPV in the qPCR assay were subject to conventional PCR and sequencing of the complete VP2 gene, as previously described [30,41].

The VP2 sequences from wild dogs were assembled and aligned, using CLC Workbench (Qiagen, Hilden, Germany), and then compared to previously characterized VP2 sequences from domestic dogs in Australia [42] and other countries, as well as reference strains of CPV, including CPV-2a-like and related viruses obtained from the GenBank database. Sequences were aligned by using the Geneious prime software package (11.0.4) using the MAFT algorithm. Phylogenetic analysis was performed using Mega X version 10.0.5 and employing the Tamura 3-parameter model of nucleotide substitution using a discrete gamma distribution *(+G)* and assuming that a certain fraction of sites is evolutionarily invariable **(***+I),* with the Nearest-Neighbor Interchange heuristic method. The Tamura 3-parameter *+G +I* model provided the best maximum likelihood (ML) fit of 24 nucleotide substitution models, with a Bayesian Information Criterion Score of 9996.026. The analysis involved 78 nucleotide sequences. Codon positions included were 1st+2nd+3rd+Noncoding. There were a total of 1719 positions in the final dataset.

### 2.5. Wild-Dog Sample Data and Owned-Dog CPV Case Occurrence Data

Data collected at the time each wild dog was culled included, sex, estimated age (from examination of dentition), date of capture, and latitude and longitude of capture location. These data were used in geospatial and statistical analysis. Eight samples missing data for date of capture or capture location were excluded from the analysis.

Retrospective data on owned-dog CPV cases occurring in the same postcodes in which wild-dog samples were collected were obtained to evaluate the potential for CPV transmission between wild- and owned-dog populations. Data from 2009 to 2015 were sourced from the Disease WatchDog^®^ database [43], a collection of national disease surveillance data for companion animals in Australia (http://www.vetcompass.com.au). Only cases that had been confirmed by diagnostic testing (fecal antigen test “ELISA”, PCR or immunofluorescence) were included in our analysis. For the purpose of geospatial and statistical analysis, only data relating to the case date (year) and postcode were used.

### 2.6. Geospatial Analysis of Wild-Dog Data and Owned-Dog Data

Mapping and geospatial analysis were performed, using ArcGIS^®^ version 10.2 (ERSI, Redlands, CA). Wild dogs’ positive and negative results were mapped by nearest postcode, which was identified from supplied latitude and longitude location data, in ArcGIS, using an ABS Postal Areas ASGS Ed 2016 Digital Boundaries Shapefile (ESRI Format) [44]. Owned-dog case occurrence was mapped by postcode. Maps were generated at the state level for SEQ and Northern NSW, and also at a regional level for Brisbane and south of Brisbane, to the border of Queensland (QLD) and NSW.

### 2.7. Statistical Analysis of Domestic-Dog CPV Cases and Association with Wild-Dog Infection

Data were analyzed by using Microsoft^®^ Excel for Mac Version 16.16.15 and Statistix^®^ version 10.0 (Analytical Software, Tallahassee, FL, USA). Odds ratios were calculated for the frequency of postcodes with wild dogs testing positive or negative for CPV and owned-dog CPV case occurrence (present or absent), for owned-dog cases occurring in the same year as wild-dog sampling, and across the entire owned dog sample period. Chi-squared (χ2) analysis was performed to test associations between wild-dog observations (present or absent) and categorical variables. For all statistical tests, a *p*-value of <0.05 was used to determine significance.

## 3. Results

### 3.1. Wild-Dog Sampling

**Figure 1:**
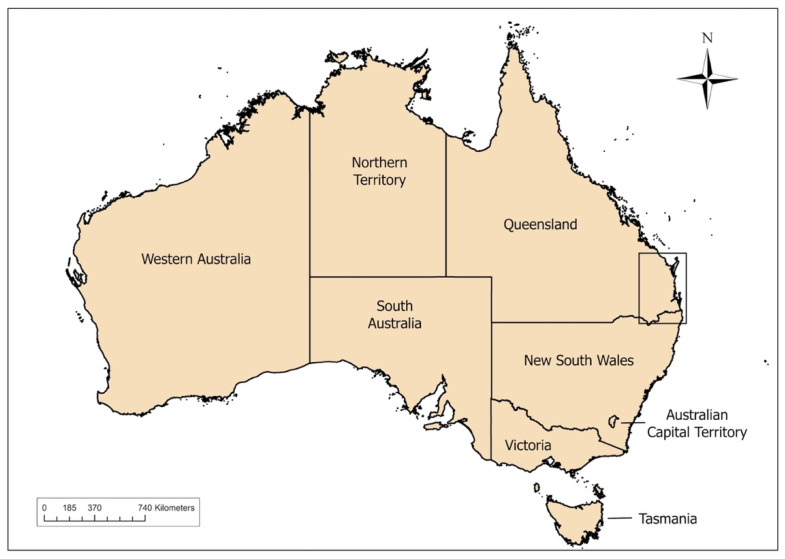
Region of wild-dog sampling (inset) between 2012 and 2015, in South East Queensland and Northeast New South Wales, Australia.

**Figure 2:**
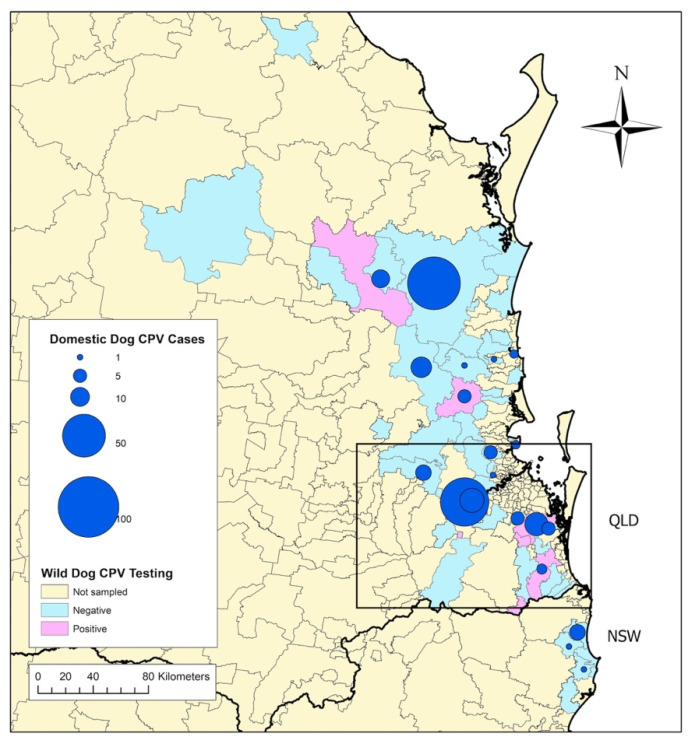
Distribution and frequency of canine parvovirus cases in domestic dogs reported by veterinarians on the Disease WatchDog^®^ national disease surveillance system between 2009 and 2015, and postcodes where wild dogs, sampled between 2012 and 2015, tested positive or negative to canine parvovirus as determined by polymerase chain reaction. Map represents South East Queensland (QLD) and Northeast New South Wales (NSW), Australia. Inset depicts area represented in Figure 3.

**Figure 3:**
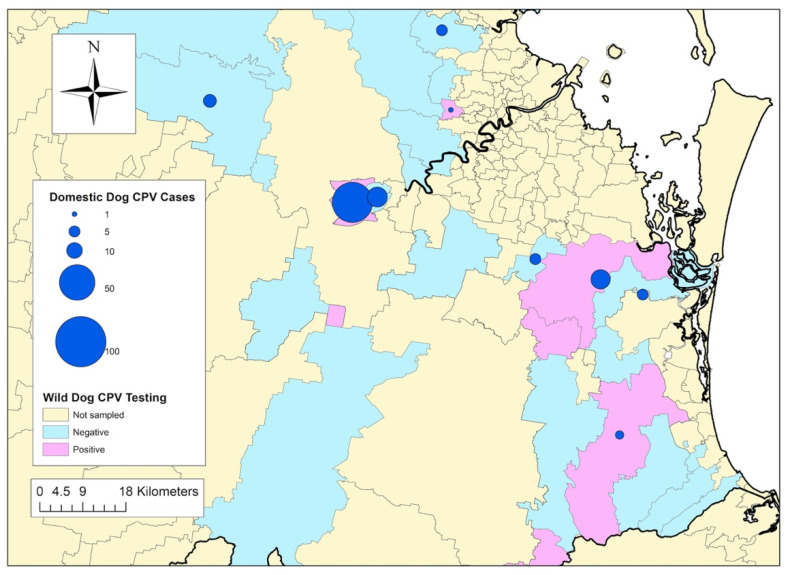
Distribution and frequency of canine parvovirus cases in domestic dogs reported by veterinarians on the Disease WatchDog^®^ national disease surveillance system between 2009 and 2015, and postcodes where wild dogs, sampled between 2012 and 2015, tested positive or negative for canine parvovirus, as determined by polymerase chain reaction. Map represents Brisbane and south of Brisbane, to the border of Queensland (QLD) and New South Wales (NSW), Australia.

Tissue samples from 171 wild dogs collected between 2012 and 2015 were available for PCR testing. Details of the cadavers sampled are reported in Table 2, and maps of the regions where wild dogs were trapped are depicted in Figure 1, Figure 2 and Figure 3.

### 3.2. DNA Detection and Quantification

Canine DNA was identified in 170/171 wild-dog cadaver samples, and CPV DNA was amplified in 4.7% (8/170) of the remaining samples analyzed. The viral load of CPV DNA in the samples ranged from 3.41 × 10^1^ to 1.95 × 10^7^ copies/µL (Table 3).

### 3.3. Geography and Prevalence of Wild-Dog Exposure to CPV

CPV DNA was amplified from eight wild dogs from seven different postcodes of QLD, but from no dogs from NSW. However, CPV detection between the states of NSW and QLD was not statistically different (χ^2^ = 1.05, df = 1, *p* = 0.3051), nor was any difference based on sex (χ^2^ = 0.08, df = 1, *p* = 0.7833), age (χ^2^ = 3.86, df = 4, *p* = 0.425) or the year in which the dogs were captured (χ^2^ = 0.91, df = 3, *p* = 0.8227) (Table 2).

### 3.4. Association between CPV Exposure in Wild Dogs and CPV Cases in Owned Dogs

In total, wild dogs were sampled from 57 different postcodes. Postcodes with one or more wild dogs testing positive to CPV were 8.63 times more likely to have reported CPV cases in owned dogs in the same year (*p* = 0.0332) and 6.43 times more likely across the entire owned-dog case sampling period (*p* = 0.0350) (Table 4).

### 3.5. Wild-Dog CPV VP2 Sequencing and Phylogenetic Analysis

**Figure 4:**
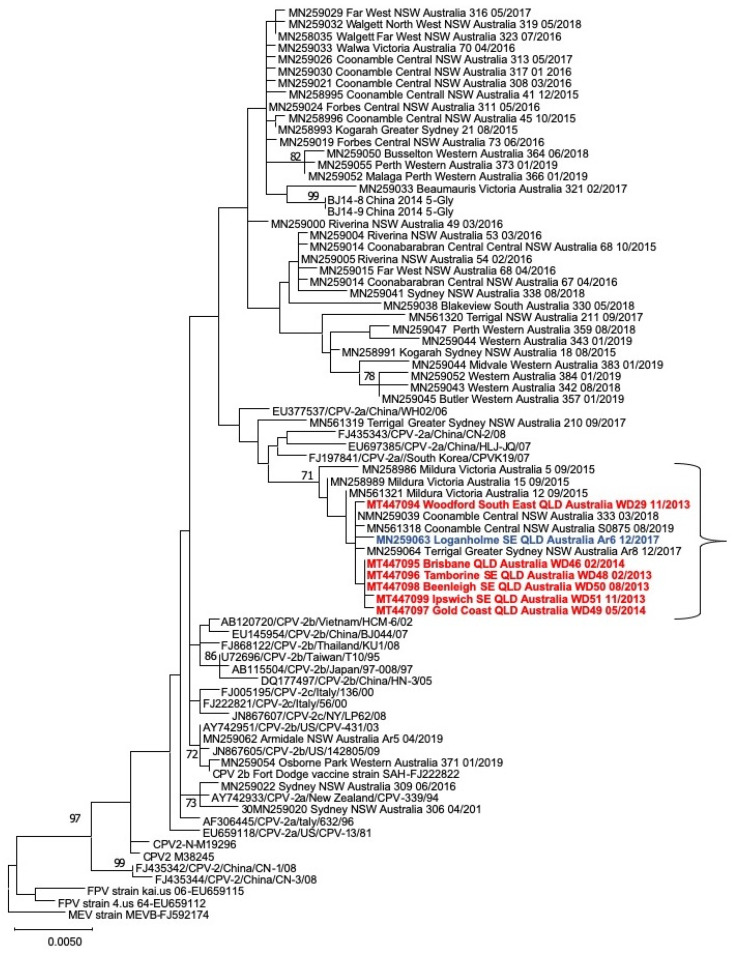
Phylogeny of CPV VP2 sequences from wild dogs in Australia (red) and a domestic dog (blue) within 10 km of the wild-dog sampling. The evolutionary history was inferred by using the Maximum Likelihood method and Tamura three-parameter model with 1000 bootstrap replicates [45]. Bootstrap values >70% are shown on the branches. The tree with the highest log likelihood (-4045.79) is shown. The tree is drawn to scale, with branch lengths measured in the number of substitutions per site.

Of the eight dogs in which CPV DNA was amplified by qPCR, VP2 sequencing was successful for six dogs and unsuccessful in the two dogs with the lowest viral loads (Table 3). Phylogenetic analysis revealed that three of the six wild-dog sequences were identical and all six were closely related, belonging to a clade of CPV-2a viruses comprising those from wild dogs, as well as viruses from dogs with parvoviral enteritis, in three different states of Australia (Victoria, NSW and QLD) (Figure 4). Viruses in this clade were characterized by the VP2 mutation (Thr-21-Ala) that differed from all other variants analyzed (Supplementary Materials Table S1). One of the viruses in this clade (GenBank accession no. MN259063) was collected from an owned dog with parvoviral enteritis, approximately 10 km from the capture location of one of the wild dogs (WD50) 3.5 years after the wild dog was culled (Figure 4 and Supplementary Materials Table S1).

## 4. Discussion

This study reports the first detection of CPV in wild-dog populations in Australia and provides phylogenetic evidence that a CPV strain from a wild-dog population was closely related to a strain from a domestic dog with parvoviral enteritis in close proximity. CPV strains from wild dogs were also closely related to viruses from other owned dogs collected over a large region of Eastern Australia. The viruses in this clade were characterized by the VP2 protein signature 21-Ala, 297-Ala, 324-Iso and 555-Val. Two of these residues (297-Ala and 555-Val) are common, well-characterized mutations among Australian CPV strains compared to the original CPV-2 virus that emerged in dogs in the late 1970s [46]. Another VP2 residue (324-Iso) in the wild-dog strains is common in Asian strains of CPV [47] and was recently identified in owned dogs in Australia [42]. The finding of this mutation in CPV strains from wild dogs further suggests introduction or circulation of Asian strains in Australia. The Thr-21-Ala mutation, present in the VP2 sequences from all of the wild dogs, was also recently identified as a novel mutation among CPVs from owned dogs in Australia [42]. The similarity of the wild-dog VP2 sequences to those from owned dogs suggests viral transmission between these two populations, although the directionality is uncertain.

Only wild dogs from SEQ and not Northern NSW tested positive for CPV, which is likely due to the smaller sample size for NSW (versus QLD), but may also reflect different risk factors for infection between these populations. Proximity to free-ranging owned dogs may be a risk factor for CPV infection in wild dogs [48]. However, our study did not investigate owned-dog population distribution or ranging behaviors, so such a transmission pathway cannot be directly assessed here. It is possible that a higher proportion of free-ranging owned dogs in an area could lead to increased infection rates of the domestic-dog population in those areas, but this requires further investigation. Close proximity to human settlements may also increase risk for CPV exposure in wild carnivores; however, previous studies have failed to demonstrate this to be statistically significant [32,36,49] or have shown no increased risk [50]. Conversely, wild-carnivore populations may have parvovirus infection cycles independent of domestic carnivores [51], and this may also be the case for some wild-dog populations in Australia; more research is needed to determine this.

No difference in age was observed between CPV-positive and -negative wild dogs, despite younger age being an identified risk factor for CPV infection and disease in unvaccinated domestic dogs [52,53] and wild canids (wolves) [54]. This may be because the method of CPV detection used in our study did not differentiate between active and recovered infections. Most (58%, 88/153) wild dogs trapped were <12 months old, which may have increased the likelihood of detecting recent or active infections and also likely reflects the shorter lifespan of wild dogs versus domestic dogs. Mortality rates from CPV in wild dogs have never been reported; however, CPV disease in young animals has been implicated in reduction of population renewal in gray wolves: the proportion of pups live-trapped each year (which had declined between 1984 and 2004) correlated with increasing CPV antibody prevalence (*r*^2^ = 0.51; *p* < 0.01) [54]. CPV might also play a role in population reduction in wild dogs in Australia. Our finding of no difference between the year that wild dogs were sampled suggests that CPV may be endemic in these populations.

We identified a significantly increased risk for domestic-dog CPV cases in geographical regions where wild-dog CPV infection was also detected. The finding of a closely related CPV strain in an owned dog collected from the same geographic region several years after detection in wild dogs suggests both low viral diversity over time and that ongoing transmission of CPV might have been occurring between the wild-dog populations in this area and neighboring owned-dog populations. Interpopulation disease transmission could be unidirectional from either population, or bidirectional [32]. Interpopulation transmission could be facilitated by roaming dogs from either or both populations entering the others’ territories and transmitting virus via fomites or directly from an infected animal through defecation. Peri-urban wild dogs in Australia have been found to have home ranges of around 17 km^2^, travel an average of 7 km/day [55] and often spend time in urban habits [56], making CPV-transmission between wild and domestic dogs likely in these areas. Fomite transmission due to human movement or other domestic/wild/feral species traversing the two territories may also occur. The ability for CPV to survive for protracted periods in the environment makes indirect transmission more likely [57].

Our detected prevalence of CPV exposure (5.3%) is much lower than that reported by serological testing of a range of wild canids in the Yellowstone National Park, USA (98%); Canadian Rocky Mountains (95%); Montana, USA (65%); Chile (49%); Portugal (38%); and Spain (17.2%) [50,58,59,60]. The true level of CPV exposure in the wild-dog population we tested is likely to be higher. However, a limitation of our study was that we only had access to wild-dog cadaver tissue, and blood for serological testing was not available. Serological testing is able to detect prior CPV exposure in individuals, with high sensitivity, due to long-lasting seropositivity following natural infection [6], whereas PCR is very sensitive to detect CPV DNA, but successful amplification depends on the availability of infected monocytes being present in the sample tissue. The viral loads obtained from six of the eight positive samples were similar to the range reported in samples collected from lymph nodes of clinically affected FPV-positive cats [30], suggesting that these six dogs were undergoing active CPV infections, while the remaining two were latently infected. Given that the epidemiological features of CPV include fecal shedding in high viral loads, environmental persistence and a high degree of contagiousness, it is likely that a larger proportion of wild dogs are exposed than are reflected in our results. The ongoing culling programs from which our samples were sourced, and the resulting reduction of wild-dog population size, may have also reduced the transmission of CPV in these populations. Serological testing of wild dogs throughout Australia is therefore warranted to determine whether actual CPV prevalence is in fact higher than our findings reflect.

## 5. Conclusions

The detection of CPV in this small population of peri-urban wild dogs suggests that parvoviral infection might be widespread among sympatric populations of wild and owned dogs in Australia. The finding of related strains of CPV in wild and owned dogs in Australia suggests that viral transmission might be occurring between these two populations, although the directionality is uncertain. It is possible that wild dogs could be responsible for some outbreaks of disease among domestic-dog populations, particularly at the urban fringe, or that infection from domestic-dog populations can spill over to wild dogs. Further research is warranted to definitively determine if CPV transmission is occurring between these populations.

## Figures and Tables

**Table 1:** Sequence and position of oligonucleotides used in the study [40] *.

Assay	Primer/Probe	Sequence 5’ - 3’	Polarity	Amplicon Size (bp)	Position ^†^
Real Time Assay	CPV-For	AAACAGGAATTAACTATACTAATATATTTA	+	93	4104–4135
	CPV-Rev	AAATTTGACCATTTGGATAAACT	−		4176–4198
	CPV-Pb	FAM—TGGTCCTTTAACTGCATTAAATAATGTACC—TAMRA	+		4143–4172

* FAM 5 6-carboxyfluorescein; TAMRA 5 6-carboxytetramethylrhodamine. ^†^ Oligonucleotide position is referred to the sequence of strain CPV-b (accession M38245).

**Table 2:** Australian wild-dog cadavers made available for PCR testing for canine parvovirus DNA, and test results, categorized by state of origin, sex, age and year trapped.

Category	Variable	No. Dogs Sampled	Percentage	No. Dogs Negative	No. Dogs Positive	Chi2	DF	*p*-Value
State	QLD	146	85.4	136	8	1.05	1	0.3051
	NSW	18	10.5	18	0			
	NR	7	4.1					
	Total	171						
Sex	Male	76	44.4	68	4	0.08	1	0.7833
	Female	87	50.9	83	4			
	NR	8	4.7					
	Total	171						
Age	<6 months	44	25.7	42	2	3.86	4	0.425
	6–12 months	44	25.7	40	3			
	1–2 years	34	19.9	33	0			
	2–5 years	14	8.2	13	1			
	>5 years	17	9.9	14	2			
	NR	18	10.5					
	Total	171						
Year captured	2012	2	1.2	2	0	0.91	3	0.8227
	2013	63	36.8	58	4			
	2014	90	52.6	85	4			
	2015	9	5.3	9	0			
	NR	7	4.1					
	Total	171						

NR = not recorded, QLD = Queensland, NSW = New South Wales, Chi2 = Chi squared statistic, DF= degrees of freedom. Seven dogs did not have corresponding location data, and sex, age and date trapped were not available for 8, 18 and 7 samples, respectively. While these were included in the PCR testing, they were excluded from the respective statistical analysis, for which their data were unavailable.

**Table 3:** Wild-dog samples testing positive for canine parvovirus DNA by quantitative PCR.

				Location Captured				
GenBank Accession No.	Sample ID	Sex	Age	Date Captured	Region	Postcode	State Locality	Viral Copies per µL/DNA
MT447094	WD29	M	<6 months	25/11/13	Woodford	4514	South East QLD	2.89 × 10^3^
MT447095	WD46	M	6–12 months	28/2/14	The Gap	4061	Brisbane QLD	2.05 × 10^4^
MT447096	WD48	F	>5 years	5/2/13	Tamborine	4270	South East QLD	1.95 × 10^7^
MT447097	WD49	M	>5 years	15/5/14	Nerang	4211	Gold Coast QLD	1.57 × 10^6^
MT447098	WD50	M	2–5 years	24/8/13	Beenleigh	4207	South East QLD	6.56 × 10^5^
MT447099	WD51	F	<6 months	27/11/13	Ipswich	4305	South East QLD	2.19 × 10^4^
NA	WD58	F	6–12 months	2/3/14	Goomeri	4601	South East QLD	4.08 × 10^1^
NA	WD59	F	6–12 months	26/2/14	The Gap	4061	Brisbane QLD	3.41 × 10^1^

QLD = Queensland, Australia; NA = not applicable.

**Table 4:** Frequency of postcodes with wild dogs testing positive or negative for canine parvovirus (CPV) and domestic-dog CPV case occurrence (present or absent). Wild-dog sampling occurred between 2012 and 2015, and domestic-dog cases were reported between 2009 and 2015.

Domestic-Dog CPV Cases	Domestic-Dog CPV Case Occurrence	Wild-Dog CPV Status				
		Positive	Negative	Total	Odds Ratio	*p*-value
In all years between 2009 and 2015	Present	5	14	19	6.43	0.0350
	Absent	2	36	38		
	Total	7	50	57		
In same year as wild-dog sampling in postcode	Present	3	4	7	8.63	0.0332
	Absent	4	46	50		
	Total	7	50	57		

## Supplementary Materials

The following are available online at https://www.mdpi.com/1999-4915/12/6/663/s1. Table S1: Amino acid substitutions in the VP2 region of strains of canine parvovirus (CPV) detected from tongue tissue of wild-dog cadavers from Northern New South Wales and South East Queensland, Australia (2013 to 2014), and in feces from dogs with confirmed parvoviral enteritis (2015 to 2019), compared to reference strains of CPV and feline parvovirus (FPV).
