# Supplementary material for: Phylogenetic and Geospatial Evidence of Canine Parvovirus Transmission between Wild Dogs and Domestic Dogs at the Urban Fringe in Australia
Source: Viruses. 2020 Jun 19;12(6):663. doi: 10.3390/v12060663 (PMC7354627; doi:10.3390/v12060663)
Supplement: Supplementary file 1 [file viruses-12-00663-s001.zip › viruses-826096-supplementary.docx]

**Supplementary Table 1.** Amino acid substitutions in the VP2 region of strains of canine parvovirus (CPV) detected from tongue tissue of wild dog cadavers from northern New South Wales and southeast Queensland, Australia (2013 to 2014), and in feces from dogs with confirmed parvoviral enteritis (2015 to 2019) (Kwan et al., 2020) [44] compared to reference strains of CPV and feline parvovirus (FPV).

| **Strain** | **Region** | **Year** | **Clade** | **5** | **21** | **80** | **87** | **92** | **93** | **99** | **101** | **103** | **178** | **232** | **252** | **262** | **267** | **297** | **300** | **305** | **321** | **323** | **324** | **339** | **373** | **375** | **413** | **426** | **555** | **564** | **568** | **570** |
| --- | --- | --- | --- | --- | --- | --- | --- | --- | --- | --- | --- | --- | --- | --- | --- | --- | --- | --- | --- | --- | --- | --- | --- | --- | --- | --- | --- | --- | --- | --- | --- | --- |
| 316 | Far West NSW | 2017 | CPV2b | G | T | R | L | F | N | D | T | A | D | I | V | A | Y | A | G | Y | N | N | Y | S | D | D | D | D | V | S | G | K |
| 319 | Far West NSW | 2018 | CPV2b | G | T | R | L | V | N | D | T | A | D | I | V | A | Y | A | G | Y | N | N | Y | S | D | D | D | D | V | S | G | K |
| 323 | Far West NSW | 2016 | CPV2b | G | T | R | L | V | N | D | T | A | D | I | V | A | Y | A | G | Y | N | N | Y | S | D | D | D | D | V | S | G | K |
| 70 | Victoria | 2016 | CPV2b | G | T | R | L | V | N | N | T | A | D | I | V | A | Y | A | G | Y | N | N | Y | S | D | D | D | D | V | S | G | K |
| 313 | Central NSW | 2017 | CPV2b | G | T | R | L | V | N | D | T | A | D | I | V | A | Y | A | G | Y | N | N | Y | S | D | D | D | D | V | S | G | K |
| 317 | Central NSW | 2016 | CPV2b | G | T | R | L | V | N | D | T | A | N | I | V | A | Y | A | G | Y | N | N | Y | S | D | D | D | D | V | S | G | K |
| 308 | Central NSW | 2016 | CPV2b | G | T | R | L | V | N | D | T | A | D | I | V | A | Y | A | G | Y | N | N | Y | S | D | D | D | D | V | S | G | K |
| 41 | Central NSW | 2015 | CPV2b | G | T | R | L | V | N | D | T | A | D | I | V | A | Y | A | G | Y | N | N | Y | S | D | D | D | D | V | S | G | K |
| 311 | Central NSW | 2016 | CPV2b | G | T | R | L | V | N | D | T | A | D | I | V | A | Y | A | G | Y | N | N | Y | S | D | D | D | D | V | S | G | K |
| 45 | Central NSW | 2015 | CPV2b | G | T | R | L | V | N | D | T | A | D | I | V | A | Y | A | G | Y | N | N | Y | S | D | D | D | D | V | S | G | K |
| 21 | Greater Sydney | 2015 | CPV2b | G | T | R | L | V | N | D | T | A | D | I | V | A | Y | A | G | Y | N | N | Y | S | D | D | D | D | V | S | G | K |
| 73 | Central NSW | 2016 | CPV2b | G | T | R | L | V | N | D | T | A | D | I | V | A | Y | A | G | Y | N | N | Y | S | D | D | D | D | V | S | G | K |
| 364 | Western Australia | 2018 | CPV2b | A | T | R | L | V | N | D | T | A | D | I | V | T | F | A | G | Y | N | N | Y | S | D | D | D | D | V | S | G | K |
| 373 | Western Australia | 2019 | CPV2b | G | T | R | L | V | N | D | T | A | D | I | V | T | Y | A | G | Y | N | N | Y | S | D | D | D | D | V | S | G | K |
| 366 | Western Australia | 2019 | CPV2b | G | T | R | L | V | N | D | T | A | D | I | V | T | Y | A | G | Y | N | N | Y | S | D | D | D | D | V | S | G | K |
| 321 | Victoria | 2018 | CPV2a | G | T | R | L | V | N | D | T | A | D | I | V | A | F | A | G | Y | N | N | I | N | N | D | D | N | V | S | G | K |
| BJ14-8 | China | 2014 | CPV2c | G | T | R | L | V | N | D | T | A | D | I | V | A | Y | A | G | Y | N | N | I | S | D | D | D | E | V | S | G | K |
| BJ-14-9 | China | 2014 | CPV2c | G | T | R | L | V | N | D | T | A | D | I | V | A | Y | A | G | Y | N | N | I | S | D | D | D | E | V | S | G | K |
| 49 | Riverina | 2016 | CPV2a | G | T | R | L | V | N | D | T | A | D | I | V | A | Y | A | G | Y | N | N | Y | S | D | D | D | N | V | S | G | K |
| 53 | Riverina | 2016 | CPV2a | G | T | R | L | V | N | D | T | A | D | I | V | A | Y | A | G | Y | N | N | Y | S | D | D | D | N | V | N | A | K |
| 48 | Central NSW | 2015 | CPV2a | G | T | R | L | V | N | D | T | A | D | I | V | A | Y | A | G | Y | N | N | Y | S | D | D | D | N | V | N | A | K |
| 54 | Riverina | 2016 | CPV2a | G | T | R | L | V | N | D | T | A | D | I | V | A | Y | A | G | Y | N | N | Y | S | D | D | D | N | V | N | A | K |
| 68 | Far West NSW | 2016 | CPV2a | G | T | R | L | V | N | D | T | A | D | I | V | A | Y | A | G | Y | N | N | Y | S | D | D | D | N | V | N | A | K |
| 338 | Greater Sydney | 2018 | CPV2a | G | T | R | L | V | N | D | T | A | D | I | V | A | Y | A | G | Y | N | N | Y | S | D | D | D | N | V | N | A | K |
| 330 | South Australia | 2018 | CPV2a | G | T | R | L | V | N | D | T | A | D | I | V | A | Y | A | G | Y | N | N | Y | S | D | D | D | N | V | S | G | K |
| 211 | Greater Sydney | 2017 | CPV2a | A | T | R | L | V | N | D | T | A | D | I | V | A | Y | A | G | Y | N | N | I | S | D | D | D | N | V | N | A | K |
| 359 | Western Australia | 2018 | CPV2a | A | T | R | M | V | N | D | T | A | D | I | V | T | Y | A | G | Y | N | N | Y | S | D | D | D | N | V | N | A | K |
| 343 | Western Australia | 2019 | CPV2a | G | T | R | M | V | N | D | T | A | D | I | V | A | F | A | G | Y | K | N | Y | S | D | D | D | N | V | N | A | K |
| 18 | Greater Sydney | 2015 | CPV2a | G | T | R | L | V | N | D | T | A | D | I | V | A | Y | A | G | Y | N | N | Y | S | D | D | D | N | V | N | A | K |
| 383 | Western Australia | 2019 | CPV2b | G | T | R | L | V | N | D | T | A | D | I | V | A | Y | A | G | D | N | D | Y | S | D | D | D | D | V | N | A | K |
| 384 | Western Australia | 2019 | CPV2b | G | T | R | L | V | N | D | T | A | D | I | V | T | Y | A | G | Y | N | N | Y | S | D | D | D | D | V | N | A | K |
| 342 | Western Australia | 2019 | CPV2a | G | T | R | L | V | N | D | T | A | D | I | V | T | F | S | G | D | N | N | Y | S | D | D | D | N | V | N | A | K |
| 357 | Western Australia | 2019 | CPV2a | G | T | R | L | V | N | D | T | A | D | I | V | T | Y | A | G | D | N | N | Y | S | D | D | D | N | V | N | A | K |
| WH02 | China | 2006 | CPV2a | A | T | R | L | V | N | D | T | A | D | I | V | A | Y | A | G | Y | N | N | I | S | D | D | D | N | V | S | G | K |
| 210 | Greater Sydney | 2017 | CPV2b | A | T | R | L | V | N | D | T | A | D | I | V | A | Y | A | G | Y | N | N | I | S | D | D | D | D | V | S | G | K |
| CN-2 | China | 2008 | CPV2a | A | T | R | L | V | N | D | T | A | D | I | V | A | F | A | G | Y | N | N | I | S | D | D | D | N | V | S | G | K |
| HLJ-JQ | China | 2007 | CPV2a | A | T | R | L | V | N | D | T | A | D | I | V | A | F | A | G | Y | N | N | I | S | D | D | D | N | V | S | G | K |
| CPVK19 | South Korea | 2007 | CPV2a | A | T | R | L | V | N | D | T | A | D | I | V | A | F | A | G | Y | N | N | I | S | D | D | D | N | V | S | G | K |
| 5 | Victoria | 2015 | CPV2a | G | A | R | L | V | N | D | T | A | D | I | V | A | F | A | G | Y | N | N | I | S | D | D | D | N | V | N | A | K |
| 15 | Victoria | 2015 | CPV2a | G | A | R | L | V | N | D | T | A | D | I | V | A | F | A | G | Y | N | N | I | S | D | D | D | N | V | S | G | K |
| 12 | Victoria | 2015 | CPV2a | G | A | R | L | V | N | D | T | A | D | I | V | A | F | A | G | Y | N | N | I | S | D | D | D | N | V | S | G | K |
| WD29 | South East QLD | 2013 | CPV2a | A | A | R | L | V | N | D | T | A | D | I | V | A | F | A | G | Y | N | N | I | S | D | D | D | N | V | S | G | K |
| 333 | Central NSW | 2018 | CPV2a | A | A | R | L | V | N | D | T | A | D | I | V | A | F | A | G | Y | N | N | I | S | D | D | D | N | V | S | G | K |
| S0875 | Central NSW | 2019 | CPV2a | A | A | R | L | V | N | D | T | A | D | I | V | A | F | A | G | Y | N | N | I | S | D | D | D | N | V | S | G | K |
| Ar6 | Queensland | 2017 | CPV2a | A | A | R | L | V | N | D | T | A | D | I | I | A | F | A | G | Y | N | N | I | S | D | D | D | N | V | S | G | K |
| Ar8 | Greater Sydney | 2017 | CPV2a | A | A | R | L | V | N | D | T | A | D | I | V | A | F | A | G | Y | N | N | I | S | D | D | D | N | V | S | G | K |
| WD46 | Brisbane QLD | 2014 | CPV2a | A | A | R | L | V | N | D | T | A | D | I | V | A | F | A | G | Y | N | N | I | S | D | D | D | N | V | S | G | K |
| WD48 | South East QLD | 2013 | CPV2a | A | A | R | L | V | N | D | T | A | D | I | V | A | F | A | G | Y | N | N | I | S | D | D | D | N | V | S | G | K |
| WD50 | South East QLD | 2013 | CPV2a | A | A | R | L | V | N | D | T | A | D | I | V | A | F | A | G | Y | N | N | I | S | D | D | D | N | V | S | G | K |
| WD51 | South East QLD | 2013 | CPV2a | A | A | R | L | V | N | D | T | A | D | I | V | A | F | A | G | Y | N | N | I | S | D | D | D | N | V | S | G | K |
| WD49 | Gold Coast QLD | 2014 | CPV2a | A | A | R | L | V | N | D | T | A | D | I | V | A | F | A | G | Y | N | N | I | S | D | D | D | N | V | S | G | K |
| HCM-6 | Vietnam | 2002 | CPV2b | A | T | R | L | V | N | D | T | A | D | I | V | A | F | A | G | Y | N | N | Y | S | D | D | D | D | V | S | G | K |
| BJ044 | China | 2007 | CPV2b | A | T | R | L | V | N | D | T | A | D | I | V | A | F | A | G | Y | N | N | Y | S | D | D | D | D | V | S | G | K |
| KU1 | Thailand | 2008 | CPV2b | A | T | R | L | V | N | D | T | A | D | I | V | A | F | A | G | Y | N | N | Y | S | D | D | D | D | V | S | G | K |
| T10 | Taiwan | 1995 | CPV2b | A | T | R | L | V | N | D | T | A | D | I | V | A | F | A | G | Y | N | N | Y | S | D | D | D | D | V | S | G | K |
| 97-008 | Japan | 1997 | CPV2b | A | T | R | L | V | N | D | T | A | D | I | V | A | F | A | G | Y | N | N | Y | S | D | D | D | D | V | S | G | K |
| HN-3 | China | 2005 | CPV2b | A | T | R | L | V | N | D | T | A | D | I | V | A | L | A | G | Y | N | N | Y | S | D | D | D | D | V | S | G | K |
| 136 | Italy | 2000 | CPV2c | A | T | R | L | V | N | D | T | A | D | I | V | A | F | A | G | Y | N | N | Y | S | D | D | D | E | V | S | G | K |
| 56 | Italy | 2000 | CPV2c | A | T | R | L | V | N | D | T | A | D | I | V | A | F | A | G | Y | N | N | Y | S | D | D | D | E | V | S | G | K |
| LP62 | NY | 2008 | CPV2c | A | T | R | L | V | N | D | T | A | D | I | V | A | F | A | G | Y | N | N | Y | S | D | D | D | E | V | S | G | K |
| CPV-431 | US | 2003 | CPV2b | A | T | R | L | V | N | D | T | A | D | I | V | A | F | A | G | Y | K | N | Y | S | D | D | D | D | V | S | G | K |
| Ar5 | North West NSW | 2019 | CPV2b | A | T | R | L | V | N | D | T | A | D | I | V | A | F | A | G | Y | K | N | Y | S | D | D | D | D | V | S | G | K |
| 142805 | US | 2009 | CPV2b | A | T | R | L | V | N | D | T | A | D | I | V | A | F | A | G | Y | K | N | Y | S | D | D | D | D | V | S | G | K |
| 371 | Western Australia | 2019 | CPV2b | A | T | R | L | V | N | D | T | A | D | I | V | A | F | A | G | Y | K | N | Y | S | D | D | N | D | V | S | G | E |
| Dodge | USA | 2003 | CPV2b | A | T | R | L | V | N | D | T | A | D | I | V | A | F | A | G | Y | K | N | Y | S | D | D | D | D | V | S | G | E |
| 309 | Greater Sydney | 2016 | CPV2a | A | T | R | L | V | N | D | T | A | D | I | V | A | F | A | G | Y | N | N | Y | S | D | D | D | N | V | S | G | K |
| CPV-339 | New Zealand | 1994 | CPV2a | A | T | R | L | V | N | D | T | A | D | I | V | A | F | A | G | Y | N | N | Y | S | D | D | D | N | V | S | G | K |
| 306 | Greater Sydney | 2016 | CPV2a | A | T | R | L | V | N | D | T | A | D | I | V | A | F | A | G | Y | N | N | Y | S | D | D | D | N | V | S | G | K |
| 632 | Italy | 1996 | CPV2a | A | T | R | L | V | N | D | T | A | D | I | V | A | F | A | G | Y | N | N | Y | S | D | D | D | N | V | S | G | K |
| CPV-13 | US | 1981 | CPV2a | A | T | R | L | V | N | D | T | A | D | I | V | A | F | S | G | Y | N | N | Y | S | D | D | D | N | V | S | G | K |
| N | USA | 1988 | CPV2 | A | T | R | M | V | N | D | I | A | D | I | V | A | F | S | A | D | N | N | Y | S | D | D | D | N | V | S | G | K |
| M38245 | USA | 1990 | CPV2 | A | T | R | M | V | N | D | I | A | D | I | V | A | F | S | A | D | N | N | Y | S | D | N | D | N | V | S | G | K |
| CN-1 | China | 2008 | CPV2 | A | T | R | M | V | K | D | I | V | D | I | V | A | F | S | A | D | N | D | Y | S | D | D | D | N | V | S | G | K |
| CN-3 | China | 2008 | CPV2 | A | T | R | M | V | K | D | I | V | D | I | V | A | F | S | A | D | N | D | Y | S | D | N | D | N | V | S | G | K |
| Kai.us-06 | USA | 2006 | FPV | A | T | K | M | V | K | D | T | V | D | V | V | A | F | S | A | D | N | D | Y | S | D | D | D | N | V | N | A | K |
| 4.us 64 | USA | 1964 | FPV | A | T | K | M | V | K | D | T | V | D | V | V | A | F | S | A | D | N | D | Y | S | D | D | D | N | V | N | A | K |
| MEVB | China | 2008 | MEV | A | T | K | M | V | K | D | I | V | D | I | V | A | F | S | V | D | N | D | Y | S | D | D | D | N | V | N | A | K |

novel mutation compared to classical CPV2 recently identified in owned Australian dogs in Australia (Kwan et al., 2020).
